# Health Records Database and Inherent Security Concerns: A Review of the Literature

**DOI:** 10.7759/cureus.30168

**Published:** 2022-10-11

**Authors:** Nduma N Basil, Solomon Ambe, Chukwuyem Ekhator, Ekokobe Fonkem

**Affiliations:** 1 Internal Medicine, Merit Health Hattiesburg, Hattiesburg, USA; 2 Neurology, Baylor Scott & White Health, McKinney, USA; 3 Neuro-Oncology, New York Institute of Technology, College of Osteopathic Medicine, Old Westbury, USA; 4 Neuro-Oncology, Baylor Scott & White Medical Center - Temple, Temple, USA

**Keywords:** protected health information, information technology, hippa, security, electronic health record (ehr)

## Abstract

The use of electronic health records (EHRs) has grown significantly in the past decade. Health information databases contain sensitive patient information, including their names and addresses, tests, diagnoses, treatment, and medical history. This information should be secured and protected from manipulation and fraudulent use by third parties. EHRs are expected to increase efficiency in healthcare delivery, improve healthcare quality, and relieve increased financial pressure. Despite these expected benefits, EHRs are potentially vulnerable to security concerns that may affect the confidentiality and privacy of patients’ personal information. This paper presents a literature review of EHRs, factors that support the security and safety of health records, potential security breaches, and solutions to inherent security concerns. The study collects data through a systematic review of past studies that have addressed the topic of EHRs and security issues, and other relevant publications on EHR systems, and procedures that help safeguard health records databases. A total of 30 sources are analyzed for all pertinent information regarding security concerns of health records databases. These sources were obtained through an internet search on credible databases, including Google Scholar, PubMed, and CINAHL databases. The results of the current study reveal the perceived vulnerability of EHRs to security concerns, common security issues, the nature of these common security concerns, Health Insurance Portability and Accountability Act rules, provider responsibilities, and recommendations for reducing EHR security risks. This paper also reveals effective strategies such as privacy-protection awareness and staff training to enhance the security of health records databases.

## Introduction and background

The United States has undergone a transition from paper to electronic health records (EHRs). The growth in EHRs has been due to widespread technology use, perceived use, and laws and regulations. The Federal Health Information Technology Strategic Plan and financial incentives introduced under the ARRA/HITECH Act (American Recovery and Reinvestment Act/Health Information Technology for Economic and Clinical Health) in 2009 have fueled the growth in EHR adoption [1]. Since the Act was passed in 2009 through 2014, approximately 75% of physician practices and 92% of hospitals have received financial incentives to adopt EHRs [1]. Bowman [2] also reports that the adoption of health information technology (HIT) and EHRs is needed to transform the United States healthcare system to be more efficient, safer, and consistent in delivering high-quality care [2].

Integrated health records are more effective, can lower costs, improve healthcare quality, and support evidence-based practice and record-keeping while facilitating sharing of information [3]. However, to maintain their effectiveness, EHRs should satisfy several requirements, including complete data, and be consistent with safety and security policies. On the other hand, as the use of EHRs grows, a large volume of data is increasingly becoming available and accessible to both authorized and unauthorized users [4]. Presently, several concerns exist about the privacy and security of healthcare data stored in electronic databases. These concerns represent some of the most critical barriers in the implementation of health records databases. For this reason, healthcare organizations should identify strategies that will help them secure EHRs and prevent them from being accessed by third parties. The focus of this literature review is to explore security issues that arise from EHR use and implementation and initiatives to mitigate these security issues.

## Review

Importance of the topic

The Health Insurance Portability and Accountability Act (HIPAA) provides federal protections for electronic patient health information by ensuring the privacy and security of identifiable health information. However, concerns over the safety of patient health information have arisen from the growth of EHRs, mobile devices, medical identity theft, and the increased exchange of data between organizations, clinicians, and federal agencies. Security breaches still occur despite the implementation of security measures that comply with HIPAA. Data breaches are also associated with substantial financial costs, and each year, they cost the United States healthcare industry about $6.5 billion, funds that could be put to better use [5]. Hence, it is essential to explore strategies that can help enhance the security of electronic databases.

Definitions and concepts

Electronic Health Records

An EHR is the electronic version of patients’ records that healthcare providers keep. Some of the information stored in EHRs includes patients’ biographical information, a catalog of patients’ symptoms, diagnosis, immunization reports, medication history, allergies, laboratory data, and radiology [4]. An EHR system can collect and store patients’ health information, and healthcare providers can share this information.

Electronic medical records (EMRs) refer to software applications used to communicate information about medical care, patient medical history, and laboratory testing outcomes [5]. Through these software applications, providers can easily access patient information instantly without time and location limitations. In addition, they can access information and get the needed support to improve clinical decision-making [6]. This increases the risk of security breaches that may threaten the privacy and confidentiality of patient information.

The purpose of implementing EHRs is to improve the delivery of quality care through a reduction of medical errors, support communication and collaboration, provide real-time patient health information, share information between clinicians, and collect health information for clinical decision-making and research purposes [7,4]. EHRs have multiple other benefits and increase the access, retrievability, and portability of patient data [8]. Compared to paper records, charts are not lost in electronic records, and the same patient record is available to multiple people simultaneously. Moreover, individual patient data can be analyzed to identify risk factors and help in guiding practice according to standards.

The Health Information Technology for Economic and Clinical Health Act

The HITECH Act was passed in 2009 to promote HIT, particularly EHRs. The Act also enhances enforcement of the HIPPA Act of 1996 by introducing stricter penalties for HIPPA compliance. Before the introduction of HITECH, only 10% of hospitals had implemented EHRs in 2008. Healthcare providers need to adopt EHRs to advance healthcare, improve care coordination, and enhance information sharing between different entities. However, because the cost of transitioning from paper to EHRs was expensive, HITECH introduced incentives to encourage EHR adoption. Following the Act’s passing, the adoption of EHRs increased from 3.2% in 2008 to 14.2% in 2015. In 2017, about 86% of office-based physicians had implemented EHRs [9]. Since the passage of the Act in 2009, there has been extensive growth in the use of electronic records. As of 2017, there was 95% usage of EMRs as a platform to document healthcare delivery and influence clinical decision-making in the United States [10]. The growth has been fueled by the need to improve healthcare quality, attain efficiency, and the increasing financial pressure.

Security and privacy of electronic health records

The most important aspects of EHR systems are privacy, security, and confidentiality [11]. Privacy refers to a moral right for individuals to determine when and how their private information is accessed and shared. The security of EHRs involves protecting data and security resources, including how data are stored and transmitted across computer systems [12]. Privacy is an aspect of security and involves enforcing rules regarding how private information is stored and shared with second and third parties. Data security is the level at which access to confidential patient information is restricted to authorized personnel only. However, a data breach may occur when data are shared with unauthorized personnel. Nonetheless, it is essential to note that privacy and security can be breached on several occasions, including unpreventable systemic identification through electronic health infrastructure and technologies. In addition, the government, healthcare workers, pharmaceutical companies, and laboratories could have valid reasons to access patient health records and, in the process, breach data security and privacy either intentionally or unintentionally [4].

Confidentiality involves protecting data from access by unauthorized people through the process of data storage, transmission, and when the patient is receiving care. Confidentiality can be attained through data encryption and controlling access to systems through the use of passwords. Confidentiality is a privacy concern, and it helps ensure patient information is protected from unauthorized editing or deletion [13]. Data availability is another commonly used term in EHR data security and privacy. It involves the ability of an authorized person to access a system and fully operate it, including gaining access to all necessary information at all times.

Privacy and security of EHR are vital because they enable patients to have trust in the decision-making capacity of the provider [14]. When patients feel like there is accuracy and confidentiality of their electronic health information, they will be more willing to disclose information to the provider [14]. When patients feel at ease sharing their health information, clinicians can get all the information they need to get a picture of the patient’s overall health and use this information to make more informed decisions [15]. However, patients withhold information or delay seeking treatment if they get the impression that information privacy and security are compromised [16]. The security of patient health records is also a concern for many patients, as the loss of sensitive health information could destroy patient trust in a hospital [17].

Health Insurance Portability and Accountability Act rules and provider responsibilities

The HIPAA is a federal law that provides nationwide protection of patient health information from being disclosed to third patients without the patient’s consent. HIPAA has a Privacy Rule that offers protection for patient-identifiable health information and a Security Rule that establishes national standards for the security of electronic Protected Health Information (ePHI) [17]. The law also has a breach notification rule that requires covered entities and business associates to notify patients of a security breach.

Healthcare providers must comply with the security, privacy, and breach notification requirements of HIPAA. Some of the health information that HIPAA protects include demographic information relating to a past, present, or future mental and physical health condition; the past, present, and future payment for healthcare services; the provision of treatment; and other individually identifiable health information such as a medical record, laboratory report, or hospital bill [18]. Those who must comply with the HIPPA requirements are healthcare providers that perform standard transactions in electronic form and billing transactions electronically, health plans, and healthcare clearinghouses.

Security concerns in electronic health databases

There are several vulnerabilities of EHRs that are putting patient safety at risk, including the following.

The Increasing Prowess of Attackers

The risk of information access by third parties is becoming more sophisticated and dangerous. Cyber attacks are rising, evasive, and almost difficult to detect [18]. The tactics and intentions of the attackers are also changing, with their intentions moving from fame to financial gains. This implies that attackers are more interested in stealing identities and bank and credit information from EHRs, and they can use this personal information for financial gains. Another worrying pattern about cyberattacks involving EHRs is that hackers use evasive technologies that make it challenging to detect security breaches. With such technologies, hackers can carry out attacks to the full extent resulting in the most damage. Besides, the expansion of vendors’ cloud-based services also means that cyberattacks are also likely to increase [19].

The Increasing Use of “Off-the-Shelf” Software Options

As EHR use grows, there is more demand for ready EHR systems by healthcare providers. To meet this demand, vendors increasingly use off-the-shelf operating systems such as Windows, Linux, Units, and similar third-party software. According to Vockley, looking at medical devices, such as patient monitors, MRI scanners, X-ray machines, and similar devices commonly used in healthcare delivery, there are a lot of similarities in the interface, and they all resemble a regular desktop and laptop [20]. This is because they are using the same operating systems that are vulnerable to the same attacks. All devices using the same operating systems can also be infected with the same viruses, thereby increasing the scope of security risks. The increase in networked medical equipment and devices implies that, if there is a security breach in the form of hacking, then traffic on the network can slow down and interfere with the delivery of healthcare services. In addition, because mobile devices are also used to access patient health information, the security issue becomes more complex [20]. The risk of patient or user harm in the case of security will often depend on the type of hazard that has occurred. For instance, medical devices that access real-time patient data through a network are more vulnerable to network disruptions.

The use of off-the-shelf products is also prone to collateral damage which can occur during security breaches. Off-the-shelf operating systems, browsers, and databases often have software patches or updates that software companies regularly develop to help protect the systems against the latest malware and security glitches. While individuals and businesses can easily install these updates and resume their work, medical technology manufacturers must first go through an FDA review process before telling their customers to install the updates to ensure that they do not interfere with the safety and functionality of the EHRs and health databases [21]. This means that operating systems and other software that support EHRs are often behind when it comes to security, making EHRs more vulnerable to security threats. The security gap can give unauthorized access to individuals with malicious intent when a security glitch occurs. Malware can be introduced to healthcare systems in various ways, including through desktops, mobile phones, and laptops that share the same network. Viruses can be easily passed on to medical devices that are not protected against malware, and the entire operating system for a hospital may shut down. According to Bowman, software bugs and viruses may interfere with data integrity, jumbling up data, deleting information [3], or putting it in the wrong place. Disorganized data may make it difficult for physicians to quickly find critical patient information, which may interfere with service delivery.

Unintended Consequences

Unintended consequences are another source of security concerns regarding EHRs. According to Graber et al., system design issues such as software design, routing of electronic data, system function or malfunction [1], and integration problems are some of the factors that threaten the security of EHRs and can adversely affect patient health outcomes. For example, a system malfunction whereby a healthcare provider cannot access patient radiology studies and delayed upload of pathology reports of adenocarcinoma are just a few examples of issues in EHRs and intended consequences that can threaten patient outcomes. This demonstrates that safety and security can be compromised when there is a malfunction in a component of the EHR system [21].

Information Sharing Concerns

Security breaches may also occur unintentionally when clinicians share information. Harman et al. report that about 73% of physicians text other physicians about patient interactions and care practices [22]. It is challenging to keep such information secure as there is no way to control what information is being shared through texting and whether third parties can intercept this information. Mobile phones are usually designed for individual use and may not have security features similar to desktops that share an organization’s network. Moreover, mobile phones can be easily misplaced or stolen, and third parties can access protected information.

When they occur, data security breaches could have significant financial and organizational harm. The impact is felt by individual hospitals, providers, business associates, and patients. It threatens the entire healthcare industry and limits the success of EHRs [23]. The most common causes of security breaches involving health records databases include the loss of unencrypted laptops and mobile phones containing patient health information. According to Chenthara et al. [24], cyberattacks involving Ransomware have more significant impacts beyond financial loss and privacy breaches. For instance, when hackers accessed the Community Health Systems (CHS) database, a known hospital group, they retrieved personal information, including the social security numbers of up to a million patients. There is also an incident whereby Anonymous, an internet vigilante group, launched a Distributed Denial-of-Service (DDoS) attack on several hospitals’ websites, which crippled medical services [24]. To avoid such attacks, healthcare providers must implement adequate safeguards in their electronic databases.

Recommendations for Reducing the Security Risks of Electronic Health Records

To ensure the security of health records databases, healthcare providers may engage physical, technical, and administrative strategies. According to Keshta and Odeh [4], administrative safeguards involve performing system audits, having an officer in charge of information technology, and developing contingency plans in case a breach occurs. The importance of administrative safeguards is that they put security procedures and policies that guide information technology use, thereby promoting the security of EHRs. On the other hand, physical safeguards involve techniques that help protect health information physically so that software and hardware are not accessed by third parties or unauthorized personnel [25]. Physical safeguards may include having security roles and secure places where servers are stored. As for technical safeguards, they aim at protecting entire information systems and the network of a healthcare institution. Compared to physical and administrative safeguards, technical safeguards are essential as most security breaches occur through electronic media such as computers and mobile phones [25]. Some of the most common technical safeguards include firewalls, data encryption, antivirus software, and cloud computing.

Physical, administrative, and technical safeguards can all be implemented to complement the security of health records databases. While physical safeguards such as physical access to servers and security cameras can prevent theft, technical safeguards such as firewalls and encryption can help prevent electronic breaches even when unauthorized personnel breaches the physical safeguards [4]. On the other hand, administrative safeguards such as comprehensive education, security plans, and having a chief information security officer can help improve the security of EHR systems and databases. Moreover, administrative features such as having manager approval of data release and employee training on handling missing data can enhance the security systems of health records databases.

Collier [26] also recommends the need to duplicate all critical hardware to enhance the security of electronic health databases. It is vital to have generators that will help support electronic systems and ensure access to patient records during power outages to prevent downtimes. There should also be comprehensive testing and monitoring strategies to ensure that patient records are readily available when needed. If a system shutdown occurs, Collier [26] recommends that paper records be made available and that there should be policies to ensure accurate patient identification. There should also be an effective communication system that does not rely on electronic systems in case information systems are compromised.

Another security option for electronic health databases is Firefox. This technology has been found to be effective in securing an organization’s network, ensuring that the existing network is protected [4]. Firefox can offer protection to the information technology systems of an organization and can be used both inside and outside the network system.

Level gateway firewalls can also be used to protect health databases. These play the role of gatekeeping in an organization’s network before the end users can access the web page. Through the gateway, the external network connections are inaccessible, which means that access to an organization’s intranet by external networks is prevented. Submission equal gateways work by blocking hackers from accessing the system directly, thus preventing access to health information. However, this technology is not commonly employed by healthcare organizations because of its complexity and cost. In this regard, it may not be applicable and viable to all healthcare organizations. There is also the network address translator, a type of firewall that effectively hides an organization’s intranet IP address so that it is not visible to external users. Network address translators create a barrier for an organization’s intranet and local area networks. While firewalls ensure the security of EHRs, all four steps of its strategies must be implemented, including service control, direction control, user control, and behavior control [4]. Before using a firewall, healthcare organizations should first perform a needs assessment, threat assessment, and budgetary assessment to identify the best possible option. Engaging all four steps of the firewall security plan is necessary to ensure the security of the entire information system [27].

Another strategy for securing EHRs is cryptography or encryption. This involves encrypting data to ensure the security of health records when it comes to exchanging information. Cryptography is clandestine writing that establishes protocols to prevent unauthorized personnel from reading secret messages [28]. The exchange of health information is subject to certain policies and specifications, and the exchange of information should be recorded when the encryptions are enabled or disabled. The HIPAA has provided guidelines through which encryption may be used to secure health information, especially when making, receiving, storing, and sharing health information. For instance, digital signatures reduce the risk of breaches when patients check their health information. However, not many people are familiar with digital signatures, resulting in their underuse [29]. The use of usernames and passwords is another form of cryptography. Users are also prompted to change passwords frequently and avoid using commonly used names and birthdates as passwords to reduce the chance of a hacker speculating the password [30]. However, this technique does not protect information theft from internal threats. This implies that employees should log out once they have completed a procedure to prevent unauthorized persons from viewing patient health information. In addition, employees must not share their identification (ID) with anyone and always log off when leaving a computer [31].

## Conclusions

The use of electronic health databases has grown exponentially in recent times and has eased the ability to share and access patient information. However, security and privacy concerns can pose a severe problem when third parties access sensitive information. With expanding demand in healthcare information storage, retrieval, and delivery process comes the need for more information security. Current laws, including HITECH and HIPAA, require EHRs to be safe and secure from access by unauthorized personnel. There are several strategies that healthcare organizations can use to enhance the security of their databases, including having a backup, use of firewalls, Firefox technology, and encryption. Multiple safeguards may be necessary to strengthen the protection of health databases.
